# Embolization of bilateral pulmonary arteriovenous fistulas: A case report

**DOI:** 10.1016/j.radcr.2026.03.067

**Published:** 2026-05-07

**Authors:** Cristian Josué Sosa Álvarez, Francisco Javier Robles Ortiz, Marisol Cervantes Rojas, Daniela Córdoba Alvarado, Oriana Yadira Mariscal Díaz

**Affiliations:** a ISSSTE Hospital Regional “Dr. Valentín Gómez Farías”, Guadalajara, Jalisco, Mexico; b Secretaría de Salud Jalisco, Guadalajara, Jalisco, Mexico

**Keywords:** Pulmonary arteriovenous malformation, Endovascular embolization, Selective pulmonary angiography, Interventional cardiology, Pulmonary fistula occlusion, Radiology case report

## Abstract

Pulmonary arteriovenous malformations (PAVMs) are rare vascular anomalies that create right-to-left shunts and may lead to hypoxemia, neurologic complications, and progressive cardiopulmonary decline. We report the case of a 56-year-old woman with chronic obstructive pulmonary disease (COPD) and a prior ischemic stroke who presented with worsening exertional dyspnea and functional decline disproportionate to spirometry findings. Contrast-enhanced chest CT revealed a rare bilateral presentation, with 2 distinct PAVMs located in the right middle lobe and the posterior basal segment of the left lung. Hemodynamic assessment showed preserved pulmonary pressures without intracardiac shunting. Selective pulmonary angiography confirmed bilateral PAVMs with significant right-to-left shunting, and both lesions were treated successfully during a single endovascular session using detachable Interlock coils. Complete angiographic occlusion was achieved without procedural complications, resulting in immediate improvement in oxygenation and clinical status. This case highlights the importance of considering PAVMs in patients with unexplained or disproportionate respiratory symptoms and underscores the effectiveness and safety of contemporary coil embolization techniques for the management of bilateral lesions in a single session.

## Introduction

Pulmonary arteriovenous malformations (PAVMs) are abnormal direct connections between the pulmonary arterial and venous systems, allowing right-to-left shunting. Their estimated prevalence ranges from 2-3 per 100,000 individuals, although they are more common in patients with hereditary hemorrhagic telangiectasia (HHT) [1]. Clinical manifestations vary and include dyspnea, cyanosis, reduced exercise tolerance, and neurological complications such as stroke or brain abscess due to paradoxical embolism. Most PAVMs are solitary; bilateral or multiple lesions are less common and may pose additional diagnostic and procedural challenges [2].

Endovascular embolization has become the preferred treatment modality, replacing surgical resection in most cases. Coils and vascular plugs are effective in closing feeding arteries ≥ 2-3 mm in diameter [[3], [4], [5]]. Bilateral disease, although uncommon, presents unique technical challenges but remains highly amenable to catheter-based therapy.

We report a case of bilateral PAVMs successfully treated in a single endovascular session using detachable coil embolization, emphasizing the role of computed tomography and selective pulmonary angiography in diagnosis and treatment.

## Case presentation

A 56-year-old woman with a 4-year history of chronic obstructive pulmonary disease (COPD) requiring long-term oxygen supplementation at 3 L/min for 16 hours per day, in addition to inhaled bronchodilator therapy consisting of ipratropium/fenoterol and budesonide/formoterol. Her medical history also included an ischemic stroke 3 years prior with minimal residual deficit (modified Rankin Score 1), for which she was receiving aspirin 100 mg daily. Over several months, she experienced a gradual decline in functional capacity, progressing from New York Heart Association class I to class III, accompanied by exertional dyspnea and a chronic morning-predominant cough. Despite optimization of inhaled therapy, her respiratory symptoms improved only partially, prompting further evaluation by the pulmonology service.

**Fig. 1:**
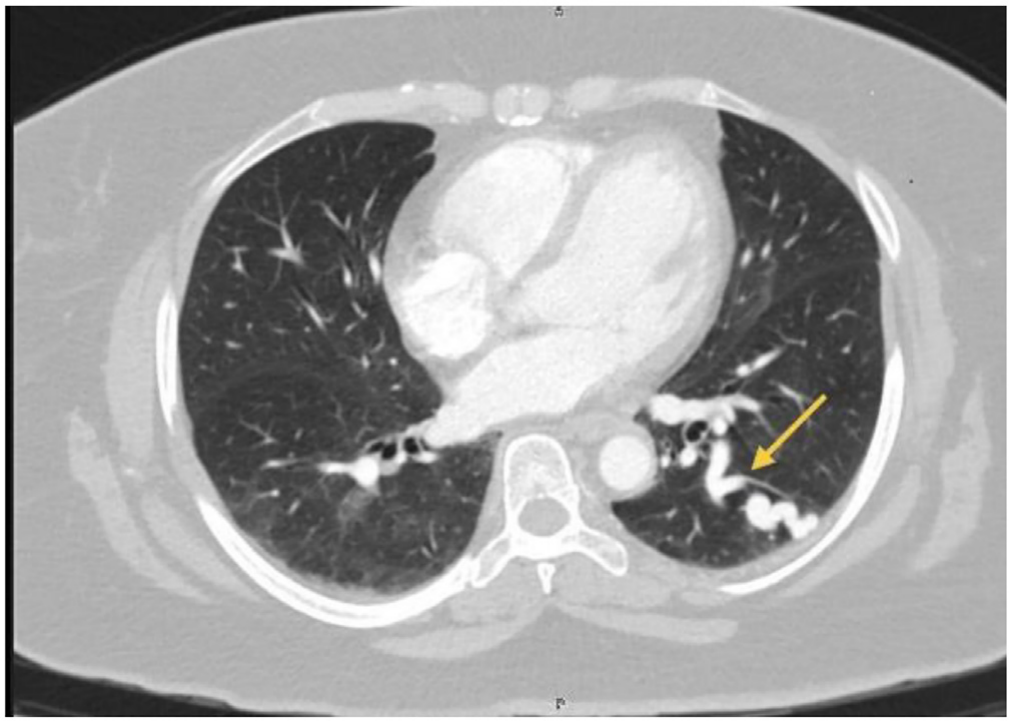
Chest CT scan with IV contrast, axial view, in the left lung, posterior basal region, shows a serpiginous structure of high attenuation following the course of a vessel (yellow arrow) with both arterial and venous dilation, consistent with a pulmonary arteriovenous malformation (arteriovenous fistula). A prominent arterial afferent vessel and a small aneurysmal sac with its corresponding efferent vein draining into the pulmonary venous circulation are observed.

**Fig. 2:**
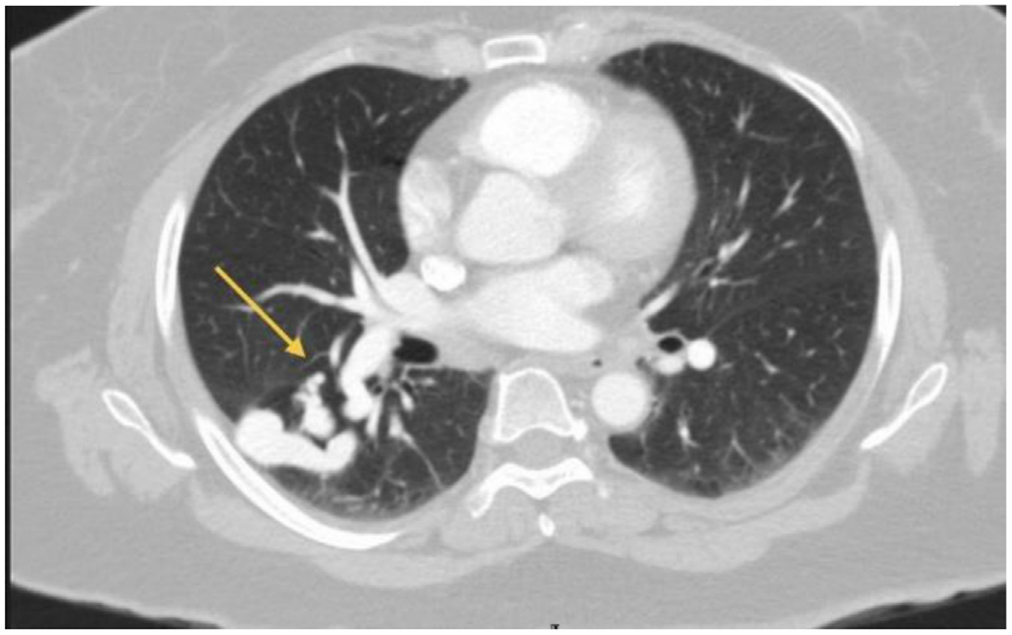
Chest CT scan with intravenous contrast, axial view, in the right middle lobe, adjacent to the medial segment, shows a serpiginous and aneurysmal vascular lesion (yellow arrow) with intense enhancement after administration of intravenous contrast, corresponding to a pulmonary arteriovenous malformation (arteriovenous fistula). A dilated arterial afferent vessel, a well-defined aneurysmal sac, and its corresponding draining vein into the pulmonary venous circulation are clearly visible in this phase.

Spirometry revealed a restrictive ventilatory pattern that did not fully correlate with the degree of her symptoms. Consequently, a contrast-enhanced computed tomography (CT) scan of the chest was obtained, revealing 2 distinct pulmonary arteriovenous malformations (PAVMs) (Fig. 1, Fig. 2). The first was located in the posterior basal segment of the left lung and appeared as a serpiginous, high-attenuation vascular structure with a dilated feeding artery, a small aneurysmal sac, and an early-opacified draining pulmonary vein. The second lesion, located in the right middle lobe, was characterized by a contrast-enhancing serpiginous vascular formation with a clearly delineated feeding artery and early venous drainage, consistent with a right-middle-lobe PAVM. These findings prompted referral to the interventional cardiology unit for definitive evaluation and management.

Right-heart catheterization demonstrated preserved pulmonary hemodynamics, with a mean pulmonary artery pressure of 19 mmHg and a right ventricular systolic pressure of 39 mmHg. Right atrial pressure measured 10 mmHg, and there was no evidence of an intracardiac shunt, as the QP/QS ratio remained within normal limits. Systemic cardiac output was 4.3 L/min. Given her progressive symptoms and the size of the feeding arteries, transcatheter embolization was indicated.

**Fig. 3:**
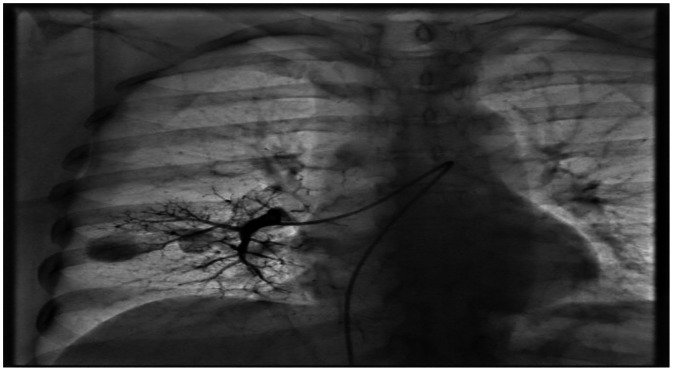
Selective right pulmonary angiography with the presence of an arteriovenous fistula in the middle lobe of the right lung that drains into the right superior pulmonary vein.

**Fig. 4:**
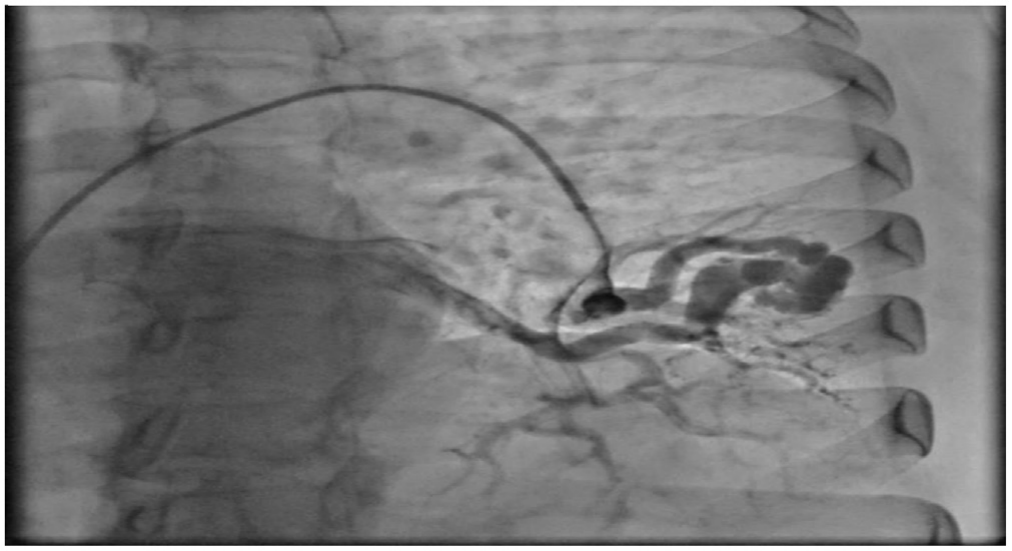
Selective left pulmonary angiography with the presence of an arteriovenous fistula in the lower lobe of the left lung that drains into the left inferior pulmonary vein.

**Fig. 5:**
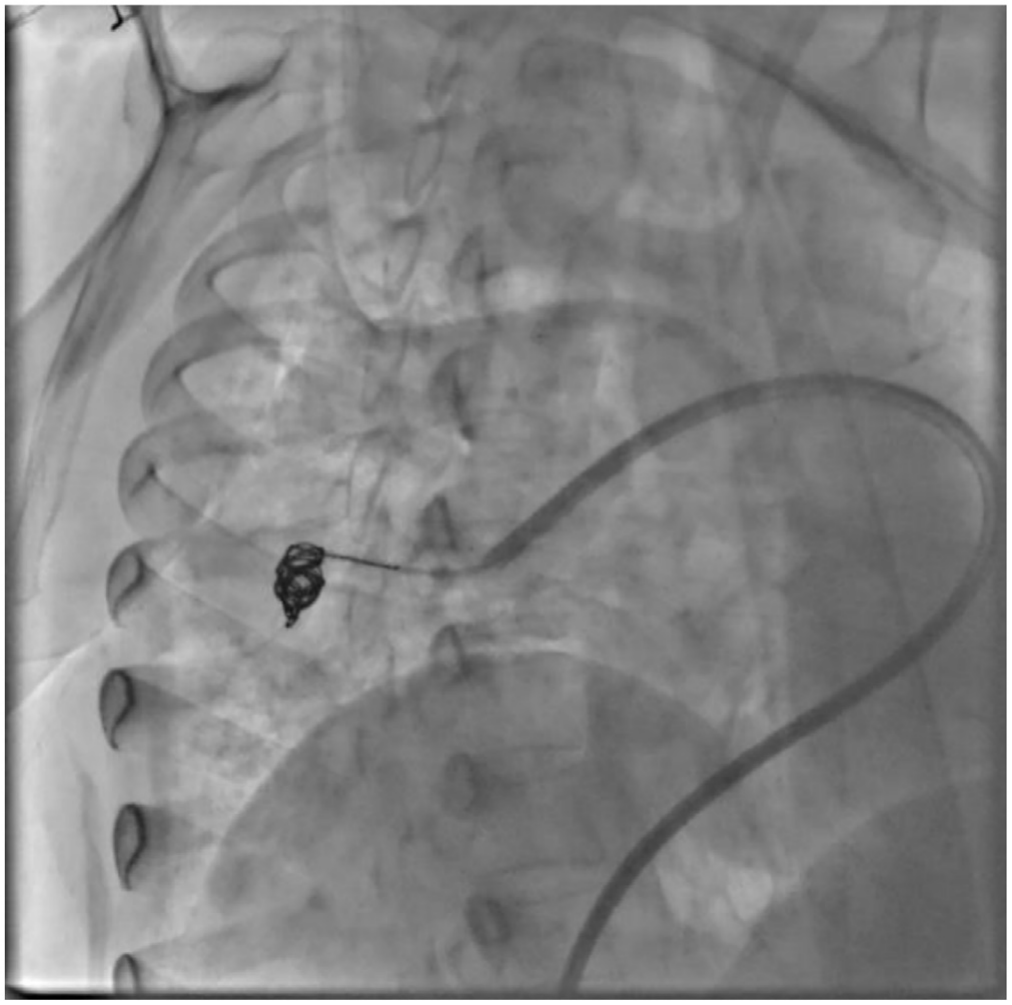
Pulmonary arteriovenous fistula embolization––left atrium of left lung.

**Fig. 6:**
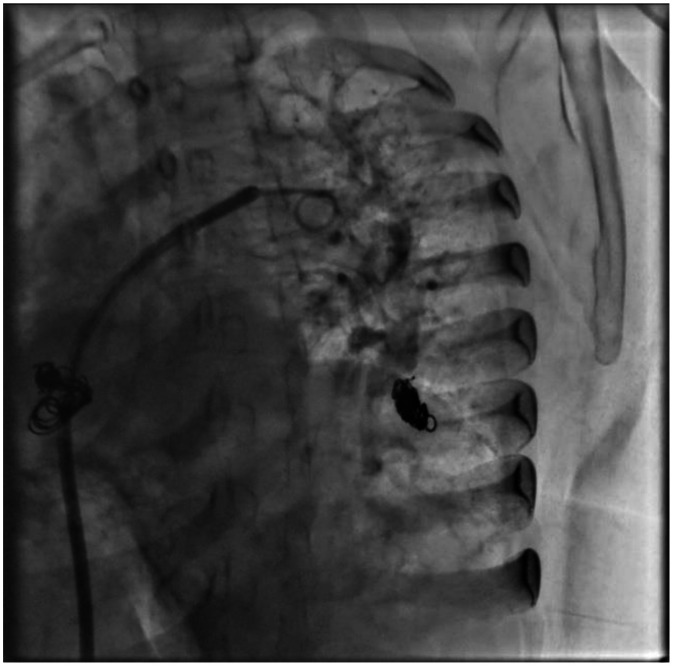
The system is advanced to the embolization site using the mother and child procedure, and embolization is performed with an 8 x 20 mm TM-35 cube interlock coil. Subsequently, a second 8 x 20 mm TM-35 2D interlock coil is advanced, followed by a third 8 x 10 mm TM-35 2D interlock coil, proximal to the previous ones. Final angiographic control was performed, without complications. Final angiography was performed using a 6 Fr pigtail catheter, showing immediate complete occlusion.

Selective right pulmonary angiography confirmed the presence of a PAVM in the right middle lobe draining into the right superior pulmonary vein (Fig. 3, Fig. 4). After establishing a mother-and-child catheter system composed of a 10 Fr Mullins sheath and a 5 Fr MP-1 catheter, the feeding artery was accessed and embolized using Interlock-35 coils measuring 10 × 40 mm and 8 × 20 mm. Final angiography demonstrated minimal residual late flow without complications. Attention was then directed to the left pulmonary vasculature, where selective angiography identified a second PAVM arising from the inferior lingular branch. The feeding artery measured 4.8 mm in diameter, with an adequate 8-mm landing zone for coil deployment. Embolization was achieved by deploying 3 Interlock-35 coils (8 × 20 mm, 8 × 20 mm 2D, and 8 × 10 mm 2D), resulting in complete and immediate angiographic occlusion of the malformation (Fig. 5, Fig. 6). The patient tolerated the procedure well and was discharged the following day with immediate improvement in oxygenation and clinical status. A 6-month follow-up CT was scheduled according to institutional post-embolization surveillance practice.

## Discussion

Pulmonary arteriovenous malformations (PAVMs) are abnormal direct communications between the pulmonary arterial and venous circulations that bypass the capillary bed, permitting unfiltered and deoxygenated venous blood to enter the systemic circulation. This pathological shunting is responsible for the cardinal manifestations of the disease, including hypoxemia, exertional dyspnea, and paradoxical embolic events such as ischemic stroke or brain abscess [1,2]. The association between PAVMs and ischemic stroke is explained by the loss of the pulmonary capillary filter, which permits thrombotic or septic emboli to bypass the lung and enter the systemic circulation. In the present case, the history of prior ischemic stroke further strengthened the indication for embolization. While the majority of PAVMs occur in association with hereditary hemorrhagic telangiectasia (HHT), sporadic cases account for a minority of presentations and often pose diagnostic challenges because their symptoms overlap with common pulmonary diseases; HHT is commonly assessed using the Curaçao criteria, which include recurrent spontaneous epistaxis, mucocutaneous telangiectasias, visceral arteriovenous malformations, and a first-degree family history of HHT. This patient did not have clinical features sufficient to support that diagnosis [1,2,5]. In this patient, progressive dyspnea that was disproportionate to spirometric findings prompted further evaluation, ultimately revealing bilateral PAVMs on contrast-enhanced CT.

Multidetector CT is considered the imaging modality of choice for diagnosing PAVMs due to its sensitivity in identifying feeding arteries, aneurysmal sacs, and early opacification of draining pulmonary veins [2,3,5]. In routine practice, PAVMs are usually detectable on standard contrast-enhanced chest CT, particularly when thin-slice multidetector acquisition is used. Dedicated radiologic assessment should focus on identifying the feeding artery, aneurysmal sac, and early draining pulmonary vein. Although no highly specialized protocol is mandatory, arterial-phase contrast enhancement and multiplanar reconstructions improve lesion conspicuity and procedural planning. Both malformations in this case displayed classic radiologic features, and the anatomic detail provided by CT enabled precise procedural planning. Treatment is recommended for symptomatic patients, those with feeding arteries measuring 2-3 mm or greater, or patients with prior neurologic complications, given the substantial risk of paradoxical embolism [1,4,5]. Although pulmonary AVMs are not routinely categorized using the same high-flow/low-flow framework applied to peripheral vascular malformations, both lesions in this case demonstrated hemodynamically significant shunting, as evidenced by dilated feeding arteries and early opacification of the draining pulmonary veins on contrast-enhanced imaging. These findings are consistent with clinically relevant lesions requiring treatment.

Endovascular embolization has become the standard of care for PAVMs and has largely replaced surgical resection. Detachable platinum coils and vascular plugs are the most commonly used devices, with success rates exceeding 90%-95% in experienced centers and durable occlusion on long-term follow-up [3,5,6]. In this patient, detachable coils were chosen because the feeding arteries had suitable diameters and landing zones for controlled deployment, while the distal and segmental anatomy favored precise placement through a stable mother-and-child catheter system. This strategy allowed progressive occlusion with preservation of control during embolization of both lesions in a single session. The mother-and-child catheter configuration used in this case allowed stable navigation of tortuous pulmonary branches and facilitated accurate coil deployment. Both lesions were successfully occluded, demonstrating the feasibility of addressing bilateral PAVMs during a single session when hemodynamic stability and anatomy permit, in line with previously reported interventional series [3,6]. The educational value of this case lies in the simultaneous treatment of bilateral lesions, the use of detachable coils in both malformations, and the integration of CT and angiographic findings to support single-session management.

The expected benefits of embolization include improved oxygen saturation, reduction in right-to-left shunting, and prevention of long-term complications, particularly neurologic events [[1], [2], [3],^6^]. Follow-up imaging, typically with contrast-enhanced CT at 6-12 months, is essential to evaluate for reperfusion or collateral recruitment, which may occur in a minority of cases [2,3,5,6]. This case emphasizes the importance of considering PAVMs in patients with unexplained dyspnea or desaturation, even in the presence of coexisting pulmonary disease, and highlights the value of multimodality imaging and modern transcatheter techniques in achieving durable therapeutic outcomes [1,2,5,6].

## Conclusion

This case demonstrates that coil embolization is a safe and effective treatment for bilateral PAVMs, providing immediate occlusion with excellent procedural outcomes. Multimodality imaging, careful vascular mapping, and the use of controlled-deployment coils are essential for successful management. Early intervention in symptomatic patients can prevent serious complications and significantly improve functional status.

## Ethical approval

Approved by the institutional ethics committee.

## Patient consent

Written informed consent was obtained from the patient for publication of this case report and accompanying images.

## References

[1] Shovlin C.L. (2014). Pulmonary arteriovenous malformations. Am J Respir Crit Care Med.

[2] Remy-Jardin M., Dumont P., Brillet P.Y. (2006). Pulmonary arteriovenous malformations treated with embolotherapy: helical CT evaluation of long-term effectiveness after 2-21-year follow-up. Radiology.

[3] Pollak J.S., Saluja S., Thabet A., Henderson K.J., Denbow N., White R.I. (2006). Clinical and anatomic outcomes after embolotherapy of pulmonary arteriovenous malformations. J Vasc Interv Radiol.

[4] Gill S.S., Roddie M.E., Shovlin C.L. (2015). Pulmonary arteriovenous malformations and their mimics. Clin Radiol.

[5] Salibe-Filho W., Rossetto de Oliveira F., Terra-Filho M. (2019). Update on pulmonary arteriovenous malformations. J Bras Pneumol.

[6] Chamarthy M.R., Park H., Sutphin P., Kumar G., Lamus D., Saboo S. (2018). Pulmonary arteriovenous malformations: endovascular therapy. Cardiovasc Diagn Ther.

